# Effects of Artificial Sweeteners on the Musculoskeletal System: A Systematic Review of Current Evidence

**DOI:** 10.3390/nu17213489

**Published:** 2025-11-06

**Authors:** Xiaoxu Xu, Qianjin Wang, Baoqi Li, Chaoran Liu, Can Cui, Ming Yi, Liting Zhai, Ronald Man Yeung Wong, Ning Zhang, Wing Hoi Cheung

**Affiliations:** 1 Musculoskeletal Research Laboratory, Department of Orthopaedics & Traumatology, The Chinese University of Hong Kong, Hong Kong, China; 2 Li Ka Shing Institute of Health Sciences, The Chinese University of Hong Kong, Hong Kong, China; 3 School of Life Sciences, The Chinese University of Hong Kong, Hong Kong, China

**Keywords:** artificial sweetener, musculoskeletal system

## Abstract

**Background:** FDA-approved artificial sweeteners (ASs) are widely used in food products due to their low-calorie content and high sweetness. However, growing evidence links them to adverse metabolic effects, including stroke and coronary heart disease. The musculoskeletal system, as a key metabolic target organ, has gradually gained attention, but the potential impact of ASs on its health remains unclear. **Objective:** This systematic review aims to assess the effects of ASs on bone and muscle, explore the underlying biological mechanisms and provide guidance for future research. **Methods:** A comprehensive literature search was conducted in PubMed, Embase, and Web of Science using relevant keywords from inception to 25 June 2025. Studies written in English, available in full text, and investigating FDA-approved ASs in relation to the musculoskeletal system were included. Two independent reviewers screened and selected the eligible studies. The findings were summarized using a narrative synthesis approach. **Results:** A total of 15 studies (12 preclinical, 3 clinical), covering aspartame, acesulfame potassium, sucralose, and saccharin were included from an initial pool of 662 articles identified across PubMed (168), Embase (368), and Web of Science (126). Among them, twelve studies focused on skeletal effects, four on muscles, and two on joints; three studies reported multiple outcomes. No studies investigated ligaments or tendons. **Conclusions:** Based on our search, this review provides a narrative synthesis of the available evidence on ASs influencing skeletal structure, development, biomechanical strength, and skeletal muscle metabolism. Potential mechanisms involve gut microbiota, oxidative stress, and signaling pathways such as SIRT1/FOXO3a and PGC-1α/UCP3. Further research is warranted to clarify these mechanisms and to assess the chronic health effects of long-term AS exposure on the musculoskeletal system in human populations.

## 1. Introduction

Sugar satisfies the primitive human craving for sweetness, a preference deeply rooted in evolutionary biology. However, with the global rise in obesity, diabetes, and other metabolic disorders, artificial sweeteners (ASs), a subgroup of non-nutritive sweeteners (NNSs), have been widely used as sugar substitutes in foods and beverages due to their high sweetness and low caloric value. Currently, the U.S. Food and Drug Administration (FDA) has approved six ASs as food additives: aspartame, sucralose, acesulfame potassium, saccharin, neotame, and advantame. These compounds are commonly found in diet sodas, sugar-free snacks, and various processed foods in our daily lives. Aspartame, for instance, has been used in more than 6000 food products worldwide [1]. In contrast to ASs, some NNSs are naturally derived, such as stevia and monk fruit, which differ in their chemical composition and metabolic pathways. These natural sweeteners are therefore not included within the scope of this review.

Although ASs are widely regarded as beneficial in weight and glycemic control, concerns about their long-term safety have persisted beyond metabolic outcomes since the 1970s, when early animal studies reported a possible link between saccharin consumption and bladder cancer [2]. In recent years, accumulating evidence has raised further concerns about potential health risks associated with ASs use. Some studies suggest that ASs may disrupt gut microbiota composition, impair glucose tolerance, and promote obesity [3,4]. In addition, ASs intake has been linked to an increased risk of stroke, ischemic stroke, coronary heart disease, and all-cause mortality [5,6].

As the major organ system of the human body, the musculoskeletal system plays a vital role in glucose homeostasis, energy balance, and endocrine regulation [7]. Its structure and function are closely linked to overall metabolic health, physical performance, and quality of life. Impairments in the musculoskeletal system are associated with increased risks of metabolic dysfunction, frailty, mobility limitations, and even mortality, particularly in older adults [8]. Despite its relevance, the musculoskeletal system has only recently been considered a target in AS-related studies, and findings in this area remain inconsistent. Some studies report neutral or even beneficial effects of ASs on muscle metabolism and bone structure [9,10,11]. whereas others report potential adverse outcomes, including altered metabolic pathways or impaired skeletal development [12,13].

Based on our search, although preliminary studies suggest that ASs may influence the musculoskeletal system, clinical evidence remains limited, and mechanistic investigations in preclinical models are still lacking. Importantly, current studies of ASs do not explicitly address potential impacts on bone health, revealing a notable gap in safety assessment. Given the critical role of the musculoskeletal system in metabolic regulation and disease risk, this systematic review aims to systematically evaluate preclinical and clinical evidence on the effects of ASs on bone and muscle, summarize potential biological mechanisms, and provide insights for future translational research and risk assessment.

## 2. Methods

### 2.1. Search Strategy

A systematic literature search was conducted to identify studies examining the effects of ASs on the musculoskeletal system. The following electronic databases were searched from inception to 25 June 2025: PubMed, Embase, and Web of Science. The search strategy combined keywords related to ASs and musculoskeletal tissues. The specific search terms used were: (aspartame OR “acesulfame potassium” OR sucralose OR neotame OR advantame OR saccharin) AND (bone OR ligament OR tendon OR cartilage OR muscle OR musculoskeletal). Additional relevant studies were identified by manually screening the reference lists of included articles and relevant reviews. The review process strictly followed the PRISMA guidelines.

### 2.2. Selection Criteria

Inclusion criteria: studies investigated the effects of the FDA-approved ASs (including aspartame, acesulfame potassium, sucralose, neotame, advantame, and saccharin) on any aspect of the musculoskeletal system (bone, ligament, tendon, cartilage, or muscle).

Exclusion criteria included: (1) Non-original research articles, such as reviews, editorials, commentaries, conference abstracts, and case reports; (2) Studies without full-text availability or not published in English; (3) Studies not reporting outcomes related to the musculoskeletal system; (4) Studies investigating non-FDA-approved sweeteners or naturally derived sweeteners; (5) Studies where artificial sweeteners were not the primary exposure of interest.

### 2.3. Study Selection

All identified articles were imported into reference management software EndNote 20, and duplicates were removed. Two independent reviewers screened the titles and abstracts for eligibility. Full texts of potentially relevant articles were retrieved and assessed for inclusion based on the criteria above. Discrepancies between reviewers were resolved by consensus or by consulting a third reviewer.

### 2.4. Data Extraction

Data was independently extracted by two reviewers using a standardized data extraction form. The following information was collected from each included study: author, year of publication, country, study design, population characteristics (including species for animal studies), type and dosage of AS, duration of exposure, outcomes measured, and main findings.

### 2.5. Data Analysis

Considering the three human clinical trials as well as animal studies with different experimental models and methods, we observed significant heterogeneity across the different datasets. Therefore, the quantitative meta-analysis synthesis was methodologically inappropriate. Thus, this analytical framework employs a narrative synthesis approach to assess evidence patterns, prioritizing thematic categorization of neuromuscular adaptation over statistical clustering.

### 2.6. Quality Assessment

Due to the substantial heterogeneity in study designs, species, and outcome measures, a formal risk-of-bias assessment was not conducted. Instead, we prioritized transparent reporting of study characteristics and outcomes in tables to facilitate qualitative comparison.

## 3. Results

**Figure 1:**
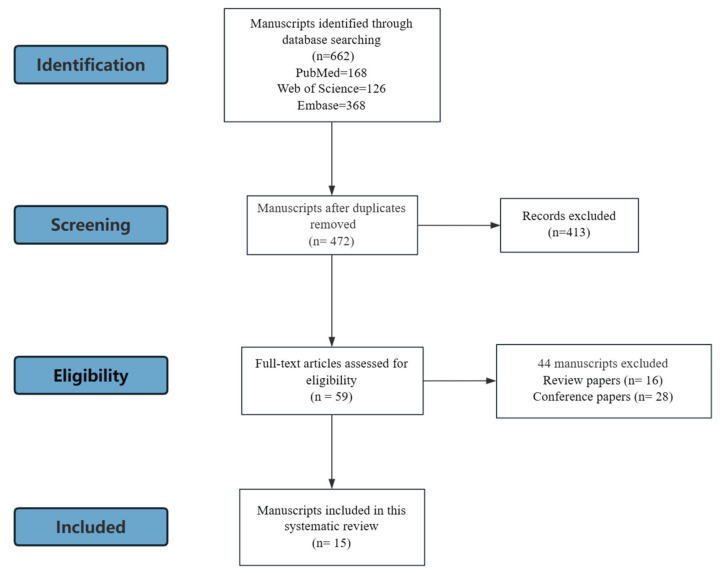
Study search and selection process.

A total of 662 records were initially identified through electronic database searches. After duplicate removal, 472 articles remained for title and abstract screening. Of these, the full texts of 59 studies were assessed for eligibility. Ultimately, 15 studies, consisting of 12 preclinical studies and 3 clinical studies, fulfilled all inclusion and exclusion criteria and were included in the final analysis (Figure 1). Among these, 12 of them discussed the impact of ASs on the skeletal system, 4 of them focused on muscle, and 2 focused on the joint. Notably, some studies reported multiple outcomes: one study addressed both bone and muscle, and two studies examined both bone and joint. No paper reported the ligament and tendon. The characteristics of the included studies, including the species or strain, sex, and age of the subjects, sweetener type used in the study, intervention and grouping, duration of the intervention, assessments are shown in Table 1, and the main results are shown in Table 2.

### 3.1. Skeletal System

Among the included studies, twelve investigated the effects of ASs on the skeletal system, including two clinical studies and ten preclinical studies. Of these, six studies focused on bone biomechanical strength, one examined skeletal developmental toxicity, and five explored the cytotoxicity and gene expression changes in bone marrow-derived mesenchymal stem cells.

#### 3.1.1. Influence of ASs on Bone Quality

A total of six articles, two clinical [14,22] and four preclinical [11,12,20,21], investigated the effects of ASs on skeletal mechanical properties, from both macroscopic and microscopic perspectives. At the macroscopic level, whole bone strength reflects the overall load-bearing capacity and fracture resistance of the bone, typically assessed through mechanical testing. At the microscopic level, parameters such as bone microarchitecture and bone mineral density (BMD), measured by micro-computed tomography (micro-CT) or dual-energy X-ray absorptiometry (DXA), provide a more detailed understanding of the bone composition and internal structure.

Two preclinical studies utilized three-point bending tests to evaluate the impact of ASs on whole bone strength. In Cyphert’s study [12], C57BL/6J mice were administered 10 g/L aspartame in drinking water starting at 1 month of age to investigate the long-term effects of aspartame on bone strength in young adult and aged mice. The young group was treated until 4 months of age, while the aged group received aspartame until 22 months. At the end of the intervention, three-point bending tests were performed to evaluate bone strength. Significant increases in whole bone strength (*p* = 0.006), were observed in the aged group compared to untreated controls. No significant effects of aspartame on bone strength were found in the young adult mice at 4 months of age. However, in another study using C57BL/6J mice, Luna et al. [11] employed a similar protocol, administering a 10 g/L blend of aspartame and acesulfame-K from 1 to 4 months of age. Three-point bending tests showed that the sweetener mixture increased whole bone strength by 39.64% after geometric adjustments (*p* < 0.001), indicating that the sweetener mixture has a positive effect on the bone strength of young mice.

Five studies [11,14,20,21,22] have examined the effects of different sweeteners on bone volume, mineral density, and trabecular or cortical microarchitecture. Two clinical studies investigated the effects of ASs on bone mass in humans [14,22]. One 6-month clinical trial conducted by Maersk et al. [22] investigated the health impact of sucrose-sweetened cola in overweight middle-aged adults. Participants were randomized into four groups, each consuming one of the following beverages daily (1 L/day): sucrose-cola, aspartame-cola, milk, or mineral water. After 6 months, DXA measurements indicated a decrease in bone mass compared to baseline across the sucrose-cola, aspartame-cola, and mineral water groups, with the greatest reduction observed in the sucrose-cola group, followed by the aspartame-cola and mineral water groups. However, the differences among these groups were not statistically significant (*p* = 0.1).

Another clinical study by Raben et al. [14] also examined the skeletal effects of sucrose and ASs in the context of energy intake and body weight in overweight adults. A total of 41 middle-aged overweight individuals were randomized to receive either a sucrose supplement or an AS supplement for 10 weeks. Whole-body DXA was used to monitor bone mineral content (BMC) and BMD. The results revealed no significant differences in BMC, bone area, or BMD between the sucrose and AS groups.

In contrast, some animal studies have suggested that ASs may have a positive impact on bone density and microstructure. Manion et al. [21] continuously administered aspartame (4 mg/kg) to STR/ORT mice from 3 weeks to 16 months of age. Whole-body radiographic scans were performed every 3–5 months under anesthesia to assess cortical bone density of the femur. Throughout the 15-month study period, mice consuming aspartame consistently exhibited higher cortical bone density compared to controls. In another study, Luna et al. [11] administered a mixture of aspartame and acesulfame-K (10 g/L) to male C57BL/6J mice starting at 4 weeks of age and continuing until 16 weeks. Micro-CT analysis of the femur showed that sweetener-treated mice had significantly higher bone volume fraction (BV/TV, *p* < 0.001) and trabecular thickness (Tb.Th, *p* < 0.001) than control mice. What is more, a significant increase in femur length in the sweetener-treated group after adjustment for geometric parameters. Parlee et al. [20] investigated the long-term skeletal impact of neonatal saccharin exposure. Neonatal C57BL/6J mice were divided into saccharin and control groups, receiving saccharin or water through maternal milk for the first 21 days after birth. At 14 weeks of age, tibial bone structure was analyzed using micro-CT. In female mice, saccharin exposure led to a reduction in total cortical bone volume, but an increase in cortical bone volume fraction and bone mineral content. In male mice, maternal saccharin exposure significantly increased trabecular bone volume (+18.2%, *p* = 0.010), bone mineral content (+25.6%, *p* = 0.008), trabecular thickness (*p* = 0.003), and cortical bone volume fraction (*p* = 0.003). Also, tibial length was found to be significantly greater in the saccharin ingested group than in the control group (*p* = 0.022).

In summary, current evidence suggests that ASs exert positive effects on bone strength, bone density and microstructure in animal experiments, while showing non-significant effects in clinical studies. Further long-term and comprehensive studies is still needed.

#### 3.1.2. Toxicity to Skeletal Development

One study indicated the toxicity of aspartame during skeletal development using the zebrafish model. Pandaram et al. [13] dissolved aspartame at concentrations of 20, 40, 60, 80, and 100 µg/mL in embryonic medium to observe the developmental toxicity of zebrafish embryos under different concentrations of aspartame. After the zebrafish embryos developed for 48 h, the embryos were fixed with 4% phosphate-buffered paraformaldehyde, stored in 100% methanol at −20 °C. Afterwards, Alcian Blue was used to stain the cartilage and incubated overnight to observe the development of cartilage and bone structures in the embryonic and larval stages. The study found that exposure to ≥40 μg/mL aspartame significantly reduced body joint formation (*p* < 0.05), axial length (*p* < 0.01), and cartilage development (*p* < 0.05) in zebrafish embryos, indicating high concentration of aspartame is detrimental to the development of animals.

#### 3.1.3. Genetic and Cellular Effects

Recent studies have shown that DNA damage occurs throughout the lifespan and is one of the major contributors to aging. In particular, DNA damage in bone marrow cells plays a critical role in disrupting bone tissue function and impairing bone homeostasis [24]. When the balance between bone resorption and bone formation is disturbed, it can lead to bone loss and even an increased risk of fractures. Therefore, it is of great significance to investigate whether ASs may affect the genetic and cellular mechanisms of the skeletal system by inducing genotoxicity, in order to better assess their skeletal safety.

A total of five articles [15,16,17,18,19] studied genetic and cellular effects of ASs on skeletal system. These studies involved three different ASs. Alsuhaibani et al. [17] evaluated the genotoxicity of aspartame at doses of 3.5, 35, and 350 mg/kg using chromosomal aberration, sister chromatid exchange (SCE), and mitotic index assays of bone marrow cells from the male Swiss Albino mice. The results showed that chromosomal aberration frequencies significantly increased at 35 and 350 mg/kg, while no significant changes were observed in SCE or the mitotic index. Mukherjee et al. [15] conducted a similar evaluation on acesulfame-K and found that doses ranging from 60 to 2250 mg/kg induced significant increases in chromosomal aberrations and the number of damaged bone marrow cells. Bandyopadhyay et al. [18] further confirmed the dose-dependent genotoxicity of aspartame, saccharin, and acesulfame-K on bone marrow cells using the single-cell gel electrophoresis (comet) assay, identifying their lowest effective doses for DNA damage as 35 mg/kg, 50 mg/kg, and 150 mg/kg, respectively.

However, in a combined toxicity study, Mukhopadhyay et al. [16] administered aspartame (3.5–350 mg/kg) together with acesulfame-K (1.5–150 mg/kg) and observed no significant effects on chromosomal aberration rates or DNA damage in the bone marrow cells of mice. These findings suggest that, within this dose range, the combination of the two sweeteners does not exert synergistic genotoxic effects. Overall, despite some variability among studies, the evidence highlights that high doses of ASs may pose genotoxic risks, providing valuable references for the evaluation of their safety thresholds. Further systematic toxicological investigations are required to determine effective doses and clarify the underlying mechanisms.

In addition to cellular toxicity analyses, Gombos et al. [19] conducted a one-week animal experiment in which CBA/CA mice were administered aspartame daily at doses of 40, 200, and 2500 mg/kg. The results demonstrated that aspartame upregulated the expression of oncogenes Ha-ras and c-myc, as well as the tumor suppressor gene p53 in bone marrow tissue, suggesting that ASs may influence gene transcriptional regulation and warrant further investigation regarding their long-term safety.

### 3.2. Muscular System

Among the included studies, four explored the effects of ASs on the muscular system, comprising two clinical and two basic research studies. Three of these focused on metabolic outcomes, while one investigated potential molecular mechanisms.

In a randomized clinical trial, Maersk et al. [22] assigned overweight participants (*n* = 47) to consume 1 L/day of one of four beverages: regular sucrose-sweetened cola, diet cola, milk, or mineral water. After six months, the intermuscular fat content of the tibialis anterior muscle was assessed using 1.5 Tesla ^1^H-Magnetic Resonance Spectroscopy(GE Medical Systems). Results showed that the diet cola group had 227% lower muscle fat content compared to the regular cola group (*p* = 0.08), though no significant difference was observed between the diet cola group and the milk or water groups.

In a prospective cohort study [23] of individuals aged 18–30 years, the intake of aspartame, saccharin, and sucralose was assessed through questionnaires at baseline, year 7, and year 20. Aspartame and sucralose intake were categorized into quintiles, while saccharin intake was categorized into tertiles. At year 25, intermuscular adipose tissue (IMAT) volume was measured using CT. This study found a positive association between higher aspartame intake and increased IMAT volume (+10.6%, *p* < 0.001). Similarly, higher saccharin consumption was also associated with greater IMAT volume (10% increase in the highest vs. lowest intake groups, *p* < 0.001), whereas sucralose intake showed no significant correlation with IMAT, suggesting a minimal impact of sucralose on muscle fat metabolism.

This finding from the 25-year longitudinal study was further supported by Malbert et al. [9], who studied obese minipigs receiving 1 mg/kg sucralose and 0.5 mg/kg acesulfame-K daily for three months. Using radiolabeled glucose tracers, they measured muscle glucose uptake and insulin sensitivity. No significant differences were observed between the ASs and control groups in glucose uptake (3.2 ± 0.12 vs. 3.2 ± 0.45 μmol·min^−1^·100 g^−1^) or insulin sensitivity (1.1 ± 0.16 vs. 1.2 ± 0.10 dL/kg·min/μU/mL × 10^−3^).

However, Santos et al. [10] reported that mice exposed to 0.03% sucralose in drinking water for 16 weeks showed significant upregulation of skeletal muscle genes involved in mitochondrial function and energy metabolism, including Ucp3, Sirt1, and Pgc1α, while there was no significant change on gastrocnemius muscle weight. These changes suggest that sucralose may influence muscle metabolic gene expression, even in the absence of overt metabolic alterations.

Overall, current evidence on the effects of ASs on the muscular system remains limited and inconclusive. While some studies suggest potential impacts on IMAT and gene expression, results are inconsistent across different sweeteners and study models. Notably, discrepancies in study duration—from short-term animal experiments to long-term clinical follow-ups—may also contribute to the variability in results, highlighting the need for further well-controlled and longitudinal investigations.

### 3.3. Joint

Two studies evaluated the effects of AS on the joint. Manion et al. [21] examined the effect of aspartame on degenerative osteoarthritis using SRT/ORT mice. The SRT/ORT mouse is a model of spontaneous osteoarthritis [25]. Researchers started feeding the mice with feed containing 4 mg/kg aspartame continuously from the age of 3 months to 18 months. During this period, the mice underwent whole-body radiological scans every 3–5 months. After the scans, the severity of osteoarthritis was evaluated by scoring radiological signs of joint damage such as osteophytes, bone deformation or bone resorption, subluxation, and swelling in the hind limbs. The final study found that the disease scores of the aspartame group were lower than those of the control group in the first 8 months. In addition, the team compared the differences in disease scores between the two groups in the early and late stages, and found that the difference in disease scores in the aspartame group was significantly better than that in the control group (*p* = 0.0047), indicating that aspartame can effectively delay the progression of osteoarthritis in mice. However, another study [13] investigating the effects of different concentrations of aspartame on cartilage development in zebrafish embryos found that when the concentration reached or exceeded 40 μg/mL, aspartame significantly inhibited cartilage formation, including joint structure and cartilage tissue development (*p* < 0.05), indicating that high concentrations of aspartame are toxic to cartilage development.

## 4. Discussion

This systematic review summarized current evidence on the effects of ASs on the musculoskeletal system, focusing on bone biomechanics, microarchitecture, growth and morphology, genetic toxicity, muscle metabolism, and joint health. The reviewed studies included both animal models and clinical participants, and involved commonly used sweeteners such as aspartame, saccharin, sucralose, and acesulfame-K. While some studies reported associations between AS exposure and musculoskeletal parameters, others found no significant effects. Overall, the findings demonstrate substantial variability depending on the sweetener type, dosage, duration of exposure, and experimental context. Despite growing public interest and widespread consumption of ASs, evidence concerning their musculoskeletal impacts remains inconclusive and sometimes contradictory.

### 4.1. Effects on the Skeletal System

#### 4.1.1. Effects on Genotoxicity

Limited studies have addressed the genotoxic potential of ASs on bone tissue. At doses ≥35 mg/kg, aspartame induces significant chromosomal aberrations in bone marrow cells [17,18] and upregulates genes related to cell cycle and apoptosis, including Ha-ras, c-Myc, and p53 [19]. Acesulfame-K at ≥60 mg/kg shows similar genotoxic profiles, suggesting that ASs may disrupt cellular homeostasis through oxidative DNA damage [15].

These effects are closely linked to oxidative stress. ASs treatment elevates reactive oxygen species (ROS), impairing mitochondrial function and promoting a feedback loop of ROS accumulation. ROS can cause DNA lesions like 8-OHdG formation and strand breaks, leading to genomic instability [26]. p53 activation under oxidative stress induces apoptosis, while c-Myc overexpression may drive abnormal proliferation and programmed cell death [27]. These changes may compromise bone marrow cell integrity and potentially disrupt normal osteogenesis.

Importantly, the metabolites of aspartame may play a central role in oxidative stress induction. Aspartame is hydrolyzed into aspartic acid (40%), phenylalanine (50%), and methanol (10%) [28]. Aspartic acid can directly activate N-methyl-D-aspartate (NMDA) receptors on neuronal synaptic membranes, leading to Ca^2+^ influx and neuronal nitric oxide synthase (nNOS) activation, thereby enhancing nitric oxide (NO) production and oxidative stress responses [29]. Methanol, on the other hand, is absorbed into the bloodstream and sequentially oxidized to formaldehyde and then formate, processes accompanied by excessive ROS generation. When this exceeds the antioxidant defense threshold, oxidative damage is triggered. Evidence has shown that ingestion of aspartame at 40 mg/kg can increase blood methanol concentrations by 3–6 fold above baseline [30], highlighting its potential role as a cytotoxic source.

Interestingly, c-Myc is also involved in physiological bone development [31]. It is expressed in proliferative chondrocytes and osteoblasts of the periosteum, regulating chondrocyte proliferation and differentiation [32]. Hence, its dysregulation may interfere with both skeletal integrity and cellular safety.

Notably, while aspartame and acesulfame-K each show genotoxicity at high doses, their combination did not produce significant DNA damage, even beyond individual toxic thresholds. This suggests a possible antagonistic interaction, warranting further mechanistic and confirmatory studies. Also, FDA has recommended the acceptable daily intake (ADI) for aspartame at 40 mg/kg body weight [33] and for acesulfame-K at 15 mg/kg body weight [34]. In contrast, the toxicological studies included in this review generally used doses far exceeding these thresholds (e.g., acesulfame-K: 15–2250 mg/kg; aspartame: 40–2500 mg/kg), substantially higher than typical human exposure levels. While such supraphysiological dosing regimens are useful for identifying potential toxicological mechanisms and safety margins, their clinical relevance remains limited. Accordingly, these findings should be regarded as exploratory evidence of possible risk signals and mechanistic pathways, rather than direct evidence of adverse effects under normal dietary consumption.

#### 4.1.2. Effects on Phenotypes

Current evidence suggests that ASs may influence bone structural integrity and mechanical properties, although results remain inconsistent and the related mechanisms remain unclear. In animal studies, long-term high-dose aspartame intake significantly improved femoral strength, cross-sectional modulus, and moment of inertia in aged male mice. However, these effects diminished after normalization for body weight, suggesting a partial dependence on weight changes [12]. In contrast, other preclinical studies have reported positive effects of ASs on cortical bone mineral density [21], trabecular thickness [11], and bone mineral content [20], indicating potential structural reinforcement under certain conditions.

Mechanistically, the positive effects of ASs on bone may be mediated via modulation of the gut microbiota. The gut hosts approximately 10^14 microbes that dynamically interact with host cells [35]. Their metabolic products, short-chain fatty acids (SCFAs), are essential in bone metabolism by lowering intestinal pH, reducing phosphate-calcium complex formation, and enhancing mineral solubility and absorption [36]. SCFAs also promote the differentiation of Treg and Th17 cells, influencing immune pathways involved in bone remodeling [37,38]. Studies have shown that ASs are poorly absorbed in the upper gastrointestinal tract and reach the colon largely intact, where they interact directly with the gut microbiota [39,40]. Several animal and human studies have shown that ASs can significantly alter the composition and metabolic activity of gut microbial communities [41,42]. Sucralose and saccharin have been reported to reduce the abundance of beneficial bacterial genera such as Bifidobacterium and Lactobacillus, while promoting the growth of pro-inflammatory taxa the abundance of Oscillospira and Ruminococcaceae, both key SCFA producers, suggesting a potential “gut–immune–bone” axis mechanism.

However, the relationship between the gut microbiota-bone axis and aging in question deserves our attention. Recent research highlights that this gut microbiota-bone axis becomes increasingly critical with aging. Age-related changes in gut microbiota typically involve a decline in beneficial SCFA-producing bacteria and an increase in pro-inflammatory taxa. These alterations are linked to chronic low-grade inflammation, impaired immune regulation, and reduced SCFA production—all of which negatively impact bone remodeling, leading to decreased bone density and compromised biomechanical properties [43]. When superimposed on the aging process, AS-induced dysbiosis may exacerbate these detrimental shifts, further diminish bone structural integrity and accelerate bone loss. Given that only one of the studies included in this paper was relevant to aging [12], it could not clearly demonstrate the impact of this potential mechanism on phenotype. This highlights the need for further studies focusing on the long-term interplay between ASs, the gut microbiota, and age-related bone health [44].

Although preclinical studies suggest that ASs may present positive effects on bone, clinical evidence remains scarce and inconsistent, with most trails reporting no significant impact on bone strength. We found that in one of the clinical studies, researchers used diet cola with aspartame and compared it with mineral water and milk. It is noteworthy that cola beverages have been reported to exert adverse effects on bone health due to their phosphoric acid and caffeine content. Therefore, the results observed in this study may be attributable to the confounding effect of cola, rather than indicating that aspartame itself has no impact on bone. Also, it is believed that the intervention durations of the two included studies [14,22]—six months and ten weeks, respectively—are relatively short compared to the human lifespan, and thus may not adequately reflect the chronic effects of long-term exposure. In addition, both studies recruited obese individuals, whose underlying metabolic disturbances may influence bone metabolism and confound the interpretation of ASs’ effects on the skeletal system. Notably, the skeletal outcome measures in these studies were relatively limited, relying solely on DXA to assess bone mass, without comprehensive evaluations of bone microarchitecture or bone metabolic markers. This limitation makes it difficult to detect early or subtle changes in skeletal health.

Therefore, future clinical studies should consider extending the intervention period, including more diverse participant populations, and employing more comprehensive and sensitive assessments of bone health to better investigate the potential effects of ASs on the skeletal system.

Some animal studies have indicated that ASs also promote linear bone growth. For example, saccharin exposure via breast milk resulted in longer tibiae in male offspring [20], while a mixture of aspartame and acesulfame-K significantly increased femur length after geometric adjustment [11]. These findings imply a possible stimulatory effect on growth plate activity, although the underlying mechanisms remain unexplored.

### 4.2. Effects on the Muscle System

Compared to the skeletal system, current evidence on the effects of ASs on skeletal muscle metabolism remains limited, and mechanistic investigations are still insufficient. Existing studies have mainly focused on changes in intermuscular adipose tissue (IMAT), a type of fat depot distributed between and around muscle fascicles [45]. Elevated IMAT has been consistently associated with insulin resistance, decreased muscle mass, and an increased risk of metabolic syndrome [46].

A long-term cohort study on obese individuals found that the intake of aspartame and saccharin was positively correlated with IMAT volume, and their consumption was also associated with increased body mass index (BMI) in a dose-dependent manner [23]. These findings align with previous reports showing a positive correlation between IMAT and total adiposity [47]. Similarly, other longitudinal studies have shown that aspartame can significantly increase BMI, suggesting potential long-term health risks [48,49]. Moreover, even after adjusting for BMI or total fat mass, IMAT remained a strong predictor of insulin sensitivity and glycemic control, indicating its independent role in metabolic regulation [50].

However, research on sucralose has not demonstrated significant associations with IMAT accumulation or changes in BMI. For instance, a 25-year cohort study by Steffen et al. found no clear link between sucralose intake and IMAT volume [23]. Nonetheless, animal experiments revealed that sucralose significantly upregulated the expression of SIRT1, PGC-1α, and UCP3 in skeletal muscle, suggesting its effects may occur through modulation of mitochondrial function rather than fat deposition [51].

SIRT1, a NAD^+^-dependent deacetylase, plays a crucial role in regulating glucose and lipid metabolism. Upregulation of SIRT1 has been shown to activate PGC-1α and FOXO1 through deacetylation, thereby alleviating mitochondrial dysfunction, insulin resistance, and obesity in mice fed a high-fat diet [52,53]. PGC-1α, as a transcriptional coactivator, can further promote UCP3 expression via activation of PPARγ or PPARδ, thus enhancing mitochondrial respiratory capacity and energy metabolism [54]. UCP3 is highly expressed in skeletal muscle mitochondria and participates in the regulation of mitochondrial membrane potential and energy homeostasis [55,56]. Although UCP3 overexpression may reduce oxidative phosphorylation efficiency, it can also facilitate glucose uptake via the PI3K pathway [57]. However, this effect appears to be context-dependent; García-Martínez et al. reported that the glucose uptake-promoting action of UCP3 was significant only under low-fat dietary conditions [58].

Research in other fields has shown that, the regulatory axis of SIRT1–PGC-1α–UCP3 becomes increasingly significant in the context of aging [59,60]. Age-related declines in SIRT1 and PGC-1α activity contribute to reduced mitochondrial biogenesis, decreased oxidative capacity, and increased susceptibility to metabolic stress and muscle atrophy [61]. The modulation of this pathway by ASs, particularly sucralose, suggests potential for both beneficial and detrimental impacts on mitochondrial function in aging muscles [62]. Given that mitochondrial dysfunction is a hallmark of muscle aging [63], any factor that perturbs mitochondrial homeostasis—such as chronic AS consumption—may have amplified effects in the elderly, potentially accelerating sarcopenic processes [64]. Conversely, targeted modulation of mitochondrial regulatory pathways by certain ASs could, under specific conditions, offer metabolic protection. Therefore, the interface between ASs, mitochondrial health, and skeletal muscle aging represents a critical area for further research. Well-designed longitudinal studies in aging models are needed to clarify whether ASs aggravates mitochondrial and metabolic decline in elderly muscle and to determine safe dietary recommendations for older adults [23].

Although these findings suggest that the SIRT1–PGC-1α–UCP3 axis may serve as a potential target through which ASs regulate muscle metabolism, current preclinical evidence remains insufficient to support this hypothesis. For example, Malbert et al. did not observe significant effects of sucralose on glucose uptake or insulin sensitivity in skeletal muscle [9], which may be due to differences in animal models, insufficient intervention duration, or small sample sizes. Likewise, Steffen et al. found no association between sucralose and IMAT levels in their long-term cohort study [23]. Therefore, these studies that failed to yield positive results also suggest that the commonly used animal models—typically young animals—may limit the observation of the true metabolic effects of ASs. Their potential impacts are more likely to be amplified or revealed in aged animals, warranting further investigation through well-designed animal experiments and mechanistic studies in the future.

### 4.3. Effects on Joint

Limited preclinical evidence suggests that ASs may offer protective effects on the joint. In a murine osteoarthritis model, aspartame intake significantly reduced arthritis scores, suggesting delayed disease progression [21]. This may be attributed to its anti-inflammatory or immunomodulatory properties. Aspartame is known to inhibit cyclooxygenase (COX-1 and COX-2), decreasing prostaglandin synthesis and thus exerting anti-inflammatory effects [65].

However, findings regarding its immune effects are conflicting. In vitro studies have shown that aspartame can increase the expression of pro-inflammatory cytokines such as TNF-α, IL-6, and IL-1β, while suppressing the anti-inflammatory cytokine IL-10 [66]. These contradictory results imply that the immunoregulatory role of aspartame may be context-dependent and influenced by dose, exposure route, and experimental model.

Conversely, evidence from embryonic models suggests potential developmental toxicity. In zebrafish embryos, exposure to ≥40 µg/mL aspartame inhibited somite formation, shaft length, and cartilage development [13]. Mechanistic analysis revealed a 40% reduction in Sirt1 expression in the aspartame group. Since Sirt1 is crucial for osteogenesis, its deficiency leads to decreased bone mass and impaired osteogenic differentiation [67]. Loss of Sirt1 in MSCs reduces both cortical and trabecular bone volume [68]. Additionally, Sirt1 can deacetylate FOXO3a to upregulate SOD2, thereby suppressing oxidative stress and promoting osteoblastic differentiation [69]. These results suggest that aspartame may impair bone morphogenesis via the Sirt1/FOXO3a signaling pathway, especially during early development.

Notably, in zebrafish embryo studies [13], researchers exposed embryos directly and continuously to varying concentrations of aspartame in the medium. This mode of exposure bypasses physiological barriers and metabolic processes, potentially exaggerating the compound’s toxicity compared to typical in vivo conditions. In contrast, in an osteoarthritis mice model [21], aspartame was administered orally at a dose of 4 mg/kg, undergoing rapid metabolism into phenylalanine, aspartic acid, and methanol. These metabolites are further processed or integrated into endogenous pathways, and the intact form of aspartame is rarely detectable in systemic circulation. Therefore, the observed biological effects in mice are likely mediated by its metabolic byproducts rather than the parent compound itself. Importantly, the concentration of these metabolites in vivo is still unclear, but probably lower than the nominal exposure concentrations used in embryo studies, which need further validation. This fundamental difference in exposure route and metabolic processing may account for the contrasting outcomes observed between zebrafish embryos and osteoarthritis mice model.

### 4.4. Limitations, Challenges and Perspectives

This review has limitations.

1. Limited number of studies

The number of included studies remains relatively small, and most were based on animal models or in vitro experiments. Given the physiological differences between zebrafish, rodents, and humans, the relevance of these findings to human musculoskeletal metabolism is uncertain, and they may not accurately reflect the structural and functional impacts on the human body.

2. Heterogeneity of study designs

Substantial heterogeneity exists among the studies in terms of AS type, dosage, mode of administration, intervention duration, and outcome measures. These inconsistencies hinder direct comparisons and limit the ability to draw definitive conclusions. In several cases, the administered doses far exceeded typical human consumption levels, potentially exaggerating the observed effects and limiting their real-world applicability.

3. Scarcity of human evidence

There is a lack of human studies specifically examining the musculoskeletal effects of ASs, especially regarding long-term or chronic exposure. While some observational cohort studies have offered preliminary insights, most lacked rigorous control of confounding variables, making it difficult to attribute musculoskeletal outcomes directly to AS intake.

Based on the limitations of the current studies, future research on the effects of ASs on the musculoskeletal system can be expanded in the following directions:

1. Conduct long-term clinical trials with reasonable doses

Future research should focus on high-quality clinical trials that use realistic, long-term exposure to reasonable doses of ASs. Compared with preclinical studies, clinical trials offer the highest level of evidence and enable a more direct and reliable evaluation of their effects on the human musculoskeletal system.

2. Conduct animal studies on long-term, low-dose exposure

Future animal studies should conduct designs involving long-term, low-dose exposure to better mimic daily dietary consumption of ASs. Such models can provide valuable insights into the chronic and cumulative effects on bone and muscle, while also allowing controlled exploration of underlying biological mechanisms that cannot be easily addressed in clinical trials.

3. Conduct studies on investigating the underlying mechanisms of ASs on musculoskeletal system

Future studies should delve deeper into the complex mechanisms underlying the interaction between ASs and the musculoskeletal system. For example, the “gut–bone axis” or “gut–muscle axis” may help elucidate whether ASs influence bone and muscle through alterations in gut microbiota or metabolic products [70,71].

4. Conduct studies related to the effects of ASs metabolites on the musculoskeletal system

Current studies on the effects of ASs on the musculoskeletal system have been limited to the sweeteners themselves, without addressing their metabolic processes in vivo. In fact, different types of AS undergo distinct metabolic pathways: some sweeteners, such as acesulfame K and saccharin, are barely metabolized and are excreted directly, while others, such as aspartame and sucralose, are broken down into multiple metabolites. Whether these metabolites may influence bone and muscle function through specific molecular mechanisms remains largely unexplored. Therefore, future research could take metabolites as a new entry point, and potential mechanisms such as mitochondrial dysfunction and oxidative stress should also be considered.

5. Conduct animal studies on age-related musculoskeletal disorders

As most existing studies have been conducted in young animal models, little is known about the effects of ASs in the context of aging. However, our review suggests that the underlying mechanisms, such as oxidative stress and SIRT1–PGC-1α–UCP3 axis, are associated with the aging process and may contribute to the development of age-related musculoskeletal disorders. Therefore, future studies should prioritize investigations using aging models, focusing on conditions such as sarcopenia, osteoporosis, and osteoarthritis, to evaluate how ASs influence bone and muscle function in elderly populations and to uncover mechanistic pathways that may differ from those in younger systems.

6. Conduct clinical translational research

Future studies should conduct standardized and clinically relevant endpoints to ensure comparability across studies and to enhance translational value. Beyond basic metabolic or molecular indicators, outcome measures should include endpoints linked to clinical significance, such as fracture incidence, bone healing quality, and muscle strength recovery. Furthermore, in the future further research may also investigate whether fracture healing may be affected, as previous preclinical studies have shown that this is compromised, especially in osteoporosis, therefore much research has been focused in accelerating this [72,73,74,75].

## 5. Conclusions

This systematic review highlights that while evidence regarding the effects of ASs on the musculoskeletal system is steadily emerging, it remains inconclusive. Current findings suggest that certain ASs compounds may affect bone structure, growth, and biomechanical properties, as well as skeletal muscle metabolism, potentially through mechanisms involving the gut microbiota, oxidative stress, and signaling pathways such as SIRT1/FOXO3a and PGC-1α/UCP3. However, these conclusions are limited by the predominance of preclinical studies and the scarcity of well-designed clinical trials.

Given the widespread and increasing global consumption of ASs, their potential impact on bone and muscle health warrants greater scientific and public health attention. Future studies should focus on well-designed randomized controlled trials to provide direct evidence, as well as mechanistic research to elucidate the underlying mechanisms affecting the musculoskeletal system and clarify the long-term consequences of ASs intake.

## Figures and Tables

**Table 1:** The characteristics of the included studies.

	Study	Strain, Species	Sex	Age	Sweetener	Intervention	Duration	Assessment
1	Raben et al. [14] (2002)	Human	Male (*n* = 6) Female (*n* = 35)	20–50 years	Aspartame Acesulfame-K Saccharin	G1: Sucrose group (*n* = 21) G2: Sweetener group (*n* = 20)	10 weeks	Bone mineral density
2	Mukherjee et al. [15] (1997)	Swiss albino mice	Male	8–10 weeks	Acesulfame-K	G1: Control (*n* = 4) G2: Acesulfame-K 15 mg/kg (*n* = 4) G3: Acesulfame-K 30 mg/kg (*n* = 4) G4: Acesulfame-K 60 mg/kg (*n* = 4) G5: Acesulfame-K 450 mg/kg (*n* = 4) G6: Acesulfame-K 1500 mg/kg (*n* = 4) G7: Acesulfame-K 2250 mg/kg (*n* = 4)	18 h	Bone marrow chromosome analysis Damaged bone marrow cells
3	Mukhopadhyay et al. [16] (2000)	Swiss albino mice	Male	8–10 weeks	Aspartame Acesulfame-K	G1: 3.5 mg/kg body weight ASPTM + 1.5 mg/kg body weight ASK G2: 35 mg/kg body weight ASPTM + 15 mg/kg body weight ASK G3: 350 mg/kg body weight ASPTM + 150 mg/kg body weight ASK	18 h	Bone marrow chromosome analysis Damaged bone marrow cells
4	Entissar S. AlSuhaibani [17] (2010)	Swiss albino mice	Male	8–10 weeks	Aspartame	G1: Control (*n* = 5) G2: 3.5 mg/kg body weight (*n* = 5) G3: 35 mg/kg body weight (*n* = 5) G4: 350 mg/kg body weight (*n* = 5)	24 h	Bone marrow chromosome analysis Sister Chromatid Exchange Assay Mitotic Indices
5	Bandyopadhyay et al. [18] (2008)	Swiss albino mice	Male	8–10 weeks	Aspartame	G1: ASP 0 mg/kg (*n* = 4) G2: ASP 7 mg/kg (*n* = 4) G3: ASP 14 mg/kg (*n* = 4) G4: ASP 28 mg/kg (*n* = 4) G5: ASP 35 mg/kg (*n* = 4)	18 h	DNA damage in bone marrow cells
					Acesulfame-K	G1: ASK 0 mg/kg (*n* = 4) G2: ASK 150 mg/kg (*n* = 4) G3: ASK 300 mg/kg (*n* = 4) G4: ASK 600 mg/kg (*n* = 4)		
					Saccharin	G1: Saccharin 0 mg/kg (*n* = 4) G2: Saccharin 50 mg/kg (*n* = 4) G3: Saccharin 100 mg/kg (*n* = 4) G4: Saccharin 200 mg/kg (*n* = 4)		
6	Gombos et al. [19] (2007)	CBA/CA mice	Female	5 weeks	Aspartame	G1: Control (*n* = 6) G2: Aspartame 40 mg/kg (*n* = 6) G3: Aspartame 200 mg/kg (*n* = 6) G4: Aspartame 2500 mg/kg (*n* = 6)	1 weeks	Bone marrow gene expression
7	Cyphert et al. [12] (2024)	C57BL/6J mice	Male (*n* = 40) Female (*n* = 40)	1 month	Aspartame	G1: chronic 10 g/L aspartame dosing from 1 to 4 months of age (*n* = 40) G2: untreated control 4 months of age (*n* = 40)	4 months	Whole bone mass Whole bone strength Femur length Tissue strength Distance to neutral axis Maximum load Yield displacement Stiffness Yield load Work to failure
			Male (*n* = 32) Female (*n* = 20)	1 month		G3: chronic aspartame dosing from 1 to 22 months of age (*n* = 22) G4: untreated control 22 months of age (*n* = 30)	22 months	
8	Luna et al. [11] (2021)	C57BL/6J mice	Male	4 weeks	Aspartame Acesulfame-K	G1: Untreated control (*n* = 10) G2: zero-calorie sweetener (*n* = 9)	12 weeks	Bone geometry Whole-bone strength Bone volume fraction Trabecular thickness Femur length
9	Parlee et al. [20] (2014)	C57BL/6J mice	Male (*n* = 23) Female (*n* = 27)	New born	Saccharin	G1: Male control (*n* = 13) G2: Male saccharin (*n* = 10) G3: Female control (*n* = 12) G4: Female saccharin (*n* = 15)	14 weeks	Tibial bone Trabecular bone Cortical bone
10	Pandaram et al. [13] (2024)	Zebra fish	/	Fertilized Eggs	Aspartame	G1: Control (*n* = 10) G2: Aspartame 20 μg/mL (*n* = 10) G3: Aspartame 40 μg/mL (*n* = 10) G4: Aspartame 60 μg/mL (*n* = 10) G5: Aspartame 80 μg/mL (*n* = 10) G6: Aspartame 100 μg/mL (*n* = 10)	24–48 h	Somite Development Cartilage development
11	Manion et al. [21] (2011)	STR/ORT mice	Male (*n* = 23) Female (*n* = 26)	3 weeks	Aspartame	G1: Regular diet (*n* = 23) G2: Aspartame 4 mg/kg containing diet (*n* = 26)	15 months	Disease score Femoral cortical bone density
12	Maersk et al. [22] (2012)	Human	Male (*n* = 17) Female (*n* = 30)	20–50 years	Aspartame	G1: sucrose-sweetened regular cola (*n* = 10) G2: aspartame sweetened diet cola (*n* = 12) G3: semiskim milk (*n* = 12) G4: still mineral water (*n* = 13)	6 months	Bone mass Intramyocellular fat
13	Steffen et al. [23] (2023)	Human	Male (*n* = 1352) Female (*n* = 1736)	18–30 years	Aspartame	Quintile 1 (*n* = 617) Quintile 2 (*n* = 618) Quintile 3 (*n* = 618) Quintile 4 (*n* = 618) Quintile 5 (*n* = 617)	25 years	Intermuscular adipose tissue
			Male (*n* = 1352) Female (*n* = 1736)	18–30 years	Saccharin	Tentile 1 (*n* = 1919) Tentile 2 (*n* = 597) Tentile 3 (*n* = 572)		
			Male (*n* = 1352) Female (*n* = 1736)	18–30 years	Sucralose	Quintile 1 (*n* = 617) Quintile 2 (*n* = 618) Quintile 3 (*n* = 618) Quintile 4 (*n* = 618) Quintile 5 (*n* = 617)		
14	Malbert et al. [9] (2019)	Yucatan mini-pigs	Male (*n* = 10) Female (*n* = 10)	3 years	Sucralose Acesulfame-K	G1: Control (*n* = 10) G2: Low-calorie sweetener diet (*n* = 10)	3 months	Glucose Uptake in Skeletal Muscle Insulin Sensitivity in Skeletal Muscle
15	Santos et al. [10] (2021)	Swiss mice	Male	6 weeks	Sucralose	G1: Control (*n* = 5) G2: Control + Sucralose (*n* = 5) G3: High-fat diet (*n* = 5) G4: High-fat diet + Sucralose (*n* = 5)	16 weeks	Gastrocnemius muscle Muscle gene expression

**Table 2:** Summary of effects of ASs on musculoskeletal outcomes.

	Study	Strain	Sweetener	Target Tissue	Direction of Effect	Results
1	Raben et al. [14] (2002)	Human	Aspartame Acesulfame-K Saccharin	Bone	Neutral	No significant changes in bone mineral content, bone area, or bone mineral density
2	Mukherjee et al. [15] (1997)	Mouse	Acesulfame-K	Bone	Negative	Significant clastogenicity at ≥60 mg/kg (*p* < 0.05).
3	Mukhopadhyay et al. [16] (2000)	Mouse	Aspartame Acesulfame-K	Bone	Neutral	No significant chromosomal aberrations (CA/cell or % damaged cells) in treated groups compared to controls.
4	Entissar S. AlSuhaibani [17] (2010)	Mouse	Aspartame	Bone	Neutral	Dose-dependent induction of chromosomal aberrations (CAs) at 35 and 350 mg/kg. No significant increase in sister chromatid exchanges (SCEs). No decrease in mitotic index (MI), indicating no cytotoxic effect on cell division.
5	Bandyopadhyay et al. [18] (2008)	Mouse	Aspartame	Bone	Negative	Dose-dependent DNA damage in bone marrow cells (>35 mg/kg)
			Acesulfame-K			Dose-dependent DNA damage in bone marrow cells >150 mg/kg)
			Saccharin			Dose-dependent DNA damage in bone marrow cells (>50 mg/kg)
6	Gombos et al. [19] (2007)	Mouse	Aspartame	Bone	Negative	Elevated oncogene (c-myc, Ha-ras) and tumor suppressor gene (p53) expression.
7	Cyphert et al. [12] (2024)	Mouse	Aspartame	Bone	Positive	Young mice/females: No significant changes. Aged males: Increased bone geometry (section modulus, cross-sectional area, moment of inertia) and strength, correlated with body mass.
8	Luna et al. [11] (2021)	Mouse	Aspartame Acesulfame-K	Bone	Positive	Increased tissue-level strength and trabecular BV/TV (+21% vs. untreated).
9	Parlee et al. [20] (2014)	Mouse	Saccharin	Bone	Positive	Males: Increased tibial length, trabecular bone volume (+35%), trabecular thickness (+13%), cortical bone volume (+11%), and bone mineral content. Females: Altered cortical bone (increased bone volume fraction and mineral content) but no trabecular changes.
10	Pandaram et al. [13] (2024)	Fish	Aspartame	Bone/Joint	Negative	Reduced cartilage development in zebrafish and delayed axial growth
11	Manion et al. [21] (2011)	Mouse	Aspartame	Bone/Joint	Positive	Aspartame significantly delayed osteoarthritis onset in STR/ORT mice compared to controls. Higher femoral cortical bone density in aspartame-fed animals.
12	Maersk et al. [22] (2012)	Human	Aspartame	Bone/Muscle	Negative	Significant increase in skeletal muscle fat in the regular cola group (117–221%, *p* < 0.05) compared to milk, diet cola, and water.
13	Steffen et al. [23] (2023)	Human	Aspartame	Muscle	Negative	Positive associations between ASs (aspartame, saccharin) and increased adipose tissue volumes.
			Saccharin			
			Sucralose			
14	Malbert et al. [9] (2019)	Pig	Sucralose Acesulfame-K	Muscle	Neutral	Skeletal muscle glucose uptake and insulin sensitivity remained unchanged in the Low-calorie sweetener diet group compared to controls.
15	Santos et al. [10] (2021)	Mouse	Sucralose	Muscle	Neutral	Upregulation of Ucp3, Sirt1, and Pgc1a in skeletal muscle of high-fat diet + Sucralose mice.
